# Effect of Diet and Supplementation on Serum Vitamin C Concentration and Antioxidant Activity in Dialysis Patients

**DOI:** 10.3390/nu15010078

**Published:** 2022-12-24

**Authors:** Anna Bogacka, Anna Sobczak-Czynsz, Edyta Balejko, Angelika Heberlej, Kazimierz Ciechanowski

**Affiliations:** 1 Department of Commodity Quality Assessment Process Engineering and Human Nutrition, Faculty of Food Sciences and Fisheries, West Pomeranian University of Technology in Szczecin, 71459 Szczecin, Poland; 2 Clinical Department of Nephrology, Transplantology and Internal Medicine, Pomeranian Medical University in Szczecin, 70111 Szczecin, Poland

**Keywords:** chronic renal failure, hemodialysis, vitamin C, FRAP, supplementation

## Abstract

Loss of vitamin C, especially in conjunction with an inadequate supply, can lead to decreased plasma concentrations of vitamin C. This in turn can lead to overt or subclinical deficiency. The present study aimed to evaluate the effects of diet and supplementation on vitamin C concentrations and serum antioxidant activity (FRAP) in hemodialysis (HD) patients. Sixty-eight HD patients participated in the study. In all of them, the diet was initially determined, and they were divided into five groups according to the diet and supplementation used. Group 1 received an unchanged diet, considered by them to be optimal; in group 2, the standard diet used in HD patients was introduced; in group 3, a standard diet enriched with natural antioxidants was employed; in group 4, a standard diet as in group 2 was used, but enriched with supplements (vitamin C, vitamin E, Se, and Zn). In contrast, group 5 consisted of HD patients with coexisting diabetes. Vitamin C serum levels were determined by high-performance liquid chromatography HPLC and antioxidant activity by The Ferric Reducing Ability of Plasma FRAP. The study shows that a well-chosen diet can slow the build-up of malnutrition and increase antioxidant activity as measured by the FRAP method in the blood of hemodialysis patients. Vitamin C supplementation can improve antioxidant status in hemodialysis patients. * The results presented in this paper complement our study, which assessed the effect of diet on the activity of erythrocyte antioxidant enzymes: Catalase (CAT), superoxide dismutase (SOD), and glutathione peroxidase (GSH-Px), but also on the concentrations of non-enzymatic antioxidants (tocopherols, carotenoids, and vitamin C) in hemodialysis patients. In the study, plasma malondialdehyde (MDA) concentrations were assessed as an indicator of oxidative damage.

## 1. Introduction

Patients with chronic renal failure (CRF) receiving renal replacement therapy have features of increased oxidative stress, with a decrease in antioxidant defense system activity [1]. The consequences of increased oxidative stress in hemodialysis (HD) patients are numerous. The following consequences have been observed: Lipid peroxidation, modification of lipoproteins, and damage to protein structures and nucleic acids. Oxidative stress also causes numerous changes at the subcellular level. It can accelerate the aging process and the development of inflammatory or degenerative diseases [2].

The hemodialysis procedure should cleanse the blood of uremic toxins. Despite the positive effect of HD, patients are at risk of increased reactive oxygen species (ROS) production. Neutrophils of dialysis patients, just after the start of dialysis, show a higher capacity to produce ROSs than cells from healthy subjects [3]. Contact of the blood with the dialysis membrane has been shown to result in increased free radical production by neutrophils [4,5,6]. Thus, mechanisms to protect cells from excessive oxidation are activated.

Vitamin C is water-soluble and is one of the most important antioxidants in the aqueous environment. The administration of ascorbic acid inhibits the expression of Nuclear-kappa B factor NF-κB in the kidney, which increases the synthesis of reactive oxygen species [7,8,9]. In addition, it plays an important role in the regeneration of α-tocopherol [10]. Studies have also found a reduction in albuminuria under the influence of vitamin C [11].

Many authors indicate increased consumption of vitamin C by hemodialysis patients and lower plasma concentrations compared to healthy individuals [12,13]. The hemodialysis treatment causes an additional reduction in blood levels of this antioxidant. It is estimated that approximately 66 mg of vitamin C passes through the dialyzer membranes into the dialysis fluid during a single hemodialysis treatment [6,14]. It is therefore suggested that supplementation in the form of a supplement to the dialysis fluid [15] or daily oral supplementation is necessary. A dose of 60–100 mg of vitamin C per day is recommended as the most appropriate in uremic patients. Higher doses may result in the accumulation of ascorbic acid metabolites and oxalates, which can lead to the formation of kidney stones, as well as the deposition of these compounds in organs, soft tissues, joints, and vascular walls [16]. In addition, ascorbic acid in high doses may exhibit pro-oxidant effects [17].

The cause of ascorbic acid deficiency may be due to both loss during the course of dialysis and a reduced supply of foods due to a fruit- and vegetable-restricted diet to prevent hyperkalemia. Saglimbene et al. [18] suggest that higher fruit and vegetable intake may reduce all-cause mortality in hemodialysis patients. Other authors postulate that the potassium supply can be reduced by eliminating fruit and vegetables from daily menus [19].

The aim of the study was to assess the effect of diet and supplementation on vitamin C concentrations and serum antioxidant activity of hemodialysis patients. In addition, the effect of the hemodialysis procedure on vitamin C concentration and antioxidant activity (FRAP) in HD subjects was assessed.

## 2. Materials and Methods

### 2.1. Characteristics of Investigated Groups

The study enrolled 68 patients with chronic renal failure (61.7 ± 11.9 years) who were treated with repeated hemodialysis for an average of 25 months (3–79 months), with a cycle of three HD treatments per week lasting 3–5 h each at the Department of Nephrology, Transplantology, and Internal Medicine, Pomeranian Medical University in Szczecin, and Stargard. Patients underwent hemodialysis using Fresenius (Homburg, Germany) units with polysulfone dialyzers. Patients were administered conventional bicarbonate dialysis fluid with the following composition: Na^+^ 138 mmol/L, K^+^ 3.0 mmol/L, Ca^2+^ 1.75 mmol/L, Mg^2+^ 0.5 mmol/L, Cl^−^ 107.5 mmol/L, and HCO^−^ 3 32 mmol/L; diabetic patients had added glucose. Blood flow through the dialyzer was constant, averaging 250 mL/min, and dialysis fluid flow was 500 mL/min.

The study was conducted over 6 months. Age and gender in the groups were not statistically significantly different.

All patients agreed to participate in the study. The study was approved by the Bioethics Committee (BN-001/118/06).

### 2.2. Study Design

1. In all patients, dietary intake (vitamin C and potassium content of diets) was initially assessed based on weekly menus recorded on dialysis and non-dialysis days.

2. Patients were divided into the following groups:

Group 1 (*n* = 6)—unchanged diet, considered by the patients to be optimal (diet A). Those in this group considered their diet sufficient for their needs and did not need the care of a dietician. Only blood was taken from patients in this group for periodic examinations.

Group 2 (*n* = 17)—the standard diet used in HD patients (diet B). The following dietary assumptions were made: Energy, 30–35 kcal/kg/day; protein, 1.0–1.2 g/body weight/day; fluid intake, 500–800 mL/day (including that contained in products) plus urine excretion; mineral supply, sodium 1800–2500 mg/day, potassium 2000–2500 mg/day, calcium up to 2000 mg/day including supplementation of calcium preparations, phosphorus 1000–1400 mg/day, and vitamin C 75–90 mg/day [20,21].

Group 3 (*n* = 25)—the standard diet used in HD patients but enriched with antioxidants naturally contained in food (diet C). The menus included additional fruit portions used interchangeably: Half a grapefruit, orange/mandarin, kiwi, and antioxidant-rich vegetables: Broccoli, spinach, and beetroot. The compote was replaced with juices: Grapefruit, blackcurrant, and orange. The changes made met daily potassium intake standards.

Group 4 (*n* = 9)—standard diet (diet B) but enriched with preparations available in pharmacies: vitamin C—100 mg/day for 6 months (Kutnowskie Zakłady Farmaceutyczne POLFA S. A., Kutno, Poland); vitamin E—300 mg/day for 6 months (Medana Pharma Terpol, Starogard Gdansk, Poland); selenium—200 μg/day 3 times a week after each hemodialysis session for 6 weeks (Walmark, Warsaw, Poland); zinc—5 mg/day for 6 months (Zincas, ZCF Farmapol, Poznan, Poland).

Group 5 (*n* = 11)—standard diet used in hemodialysis patients with diabetes (diet B).

3. Groups 2–5 were happy to receive nutritional education and agreed to work with a dietician. During dialysis with each patient, the diet was analyzed individually. During the 6 months of the study, individual weekly menus were prepared for the patients in these groups.

4. During the 6-month follow-up, blood was periodically drawn from each patient for biochemical tests (at months 0, 3, and 6).

### 2.3. Serum Preparation

Venous blood was collected from an established arteriovenous fistula at months 0, 3, and 6 from the start of the diet and immediately before and after the dialysis procedure. Samples were centrifuged at 2000× *g* at 4 °C for 15 min to separate serum from blood cells. Serum vitamin C concentration and antioxidant activity (FRAP) were determined.

### 2.4. Biochemical Analysis

Serum vitamin C concentration was determined by high-performance liquid chromatography HPLC [22], using the reduction of dehydroascorbic acid to ascorbic acid with a reducing compound, namely, dithiothreitol. Analyses were performed using a liquid chromatograph from Agilent Technologies 1200 Series, CA, USA. In two tubes, 300 µL each of serum was placed. First, 10 mL of the dithiothreitol solution was added to reduce dehydroascorbic acid to ascorbic acid. The tubes were protected from light at room temperature. A 30 mg/L aqueous solution of 4-hydroxyacetanilide was then added to both tubes, successively dispensing demineralized water to one tube where the reduction of dehydroascorbic acid took place and demineralized water and 8% metaphosphoric acid to the other tube to deproteinize the samples. The samples were left in a dark place at room temperature, then centrifuged at 13,100 rpm for 10 min at 4 °C and fed onto the chromatography column. Then, 20 µL of the supernatant was taken. Readings were taken at 245 nm using a diode array detector (DAD) (Agilent Technologies, CA, USA). Sample separation was carried out on an Eclipse XDB-C 18 5 µm column (Perlan Technologies, Warsaw, Poland), at 23 °C. A mixture of the H_2_O/acetonitrile/ KH_2_PO_4_ (1.0 M)/H_3_PO_4_ solution (68%) at the ratio of 913.5/75/10/1.5 was used as the mobile phase. The flow rate of the mobile phase was 0.475 mL/min. The total vitamin C content was the sum of ascorbic acid and dehydroascorbic acid concentrations using a standard curve.

Antioxidant activity was determined by the FRAP (The Ferric Reducing Ability of Plasma) method according to Benzie and Strain [23] in blood serum. The method is based on the ability of certain antioxidants to reduce Fe^+3^ to Fe^+2^. In the FRAP mixture, the reagent TPTZ (2,4,6-tripyridyl-s-triazine) forms a blue complex with Fe^+3^. The increase in absorbance of the complex is proportional to the number of antioxidants present in the sample. The reaction conditions, mainly pH 3.6 of the environment and a temperature of 37 °C, facilitate its formation and prevent tissue iron from participating in the reaction. The measurement was performed on a Perkin Elmer UV/VIS Lambda 20 (Perlan Technologies Polska Sp. z o. o., Warsaw, Poland) spectrophotometer at 593 nm. After zeroing the instrument on acetate buffer, the actual measurements proceeded with quartz glass cuvettes with an optical length of 1 cm. To the cuvettes, 3 mL of the FRAP reagent was added, and an additional 100 µL of plasma was added to the measuring cuvette. The sample thus prepared was left at room temperature for 30 min.

A calibration curve was prepared using the Trolox reagent (Merck KGaA, Darmstadt, Germany). Results are presented in units of Mm Trolox/L.

### 2.5. Statistical Analysis

All results are presented as the arithmetic mean ± standard deviation (SD). The results were statistically analyzed using Statistica 13 (Statsoft Polska Sp. z o.o., Krakow, Poland). ANOVA analysis showed that there was no normal distribution of the studied characteristics, therefore the Tukey post-hoc test was used to test the significance of the differences, assuming a significance level of *p* < 0.05. Due to the large number of results concerning statistically significant differences, it was decided not to include them in the table, and the most important ones are described in the “Results” section. In addition, the correlation between vitamin C concentration and FRAP was also investigated.

## 3. Results

### 3.1. Biochemical Measurements

Sixty-eight clinically stable patients with a mean age of 61.7 ± 11.9 years (40–79 years) maintained on hemodialysis for a mean period of 25.0 ± 19.9 months were enrolled in this study. The values of the liver function tests before and after dialysis were not significantly different between groups. Some of these data are summarized in Table 1. The level of urea was significantly reduced after dialysis (average in groups 134.8 ± 29.3 vs. 45.5 ± 12.6 mg/dL).

### 3.2. Nutritional Status Assessment

The highest mean body mass index (BMI) values were recorded in group 3 at both the start and end of the study (Table 2). At the start of the study, the weight of these patients was at the upper end of the normal BMI range and remained constant thereafter. Assuming a BMI range of 18.5–24.9 as normal and 25.0–29.9 as overweight, there were no decreases in weight or excessive height during follow-up in the groups fed under dietitian supervision. In contrast, group 1 showed a significant decrease in BMI during the 6-month follow-up.

Thus, it has been shown that the use of a well-balanced diet can influence the nutritional status of hemodialysis patients, either through a slight increase in body mass index or by maintaining baseline values.

**Figure 1:**
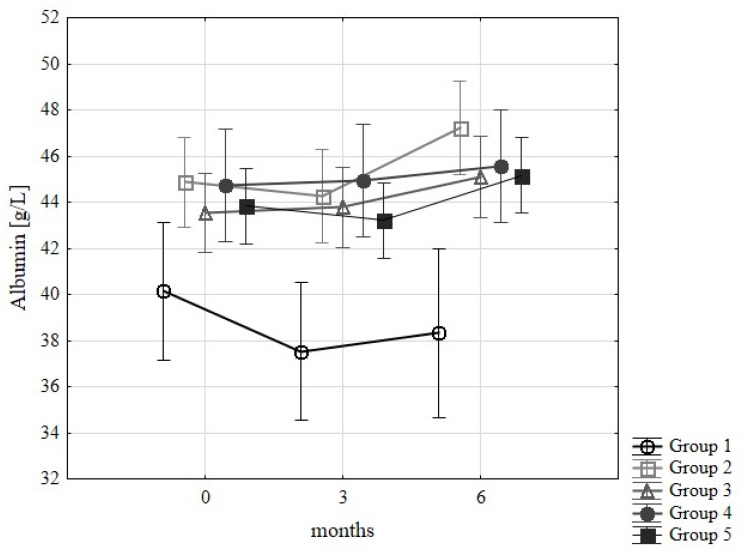
Changes in albumin levels (g/L) in examined groups of patients during 6 months of implementation of diet recommendations.

One of the biochemical exponents of increasing malnutrition may be reduced values of the albumin concentration (Figure 1). The norm was a concentration of 35–50 g/L, and values of 35–39 g/L were taken as the lower limit of normal with 40–46 g/L as the middle limit.

Albumin concentration was affected only by the type of group. The lowest plasma albumin content was observed in group 1, higher values were observed in groups 3 and 5, and the highest was seen in groups 2 and 4. These were baseline values, and the differences were not statistically significant.

Statistical analysis showed significant differences between groups 1 and 4 in month 3 of the study and between groups 1 and 2 in month 6.

In group 1, the albumin concentration decreased after 6 months of the study. This was not the case in the other groups that followed the principles of proper nutrition, where an increase in the albumin concentration was observed.

**Figure 2:**
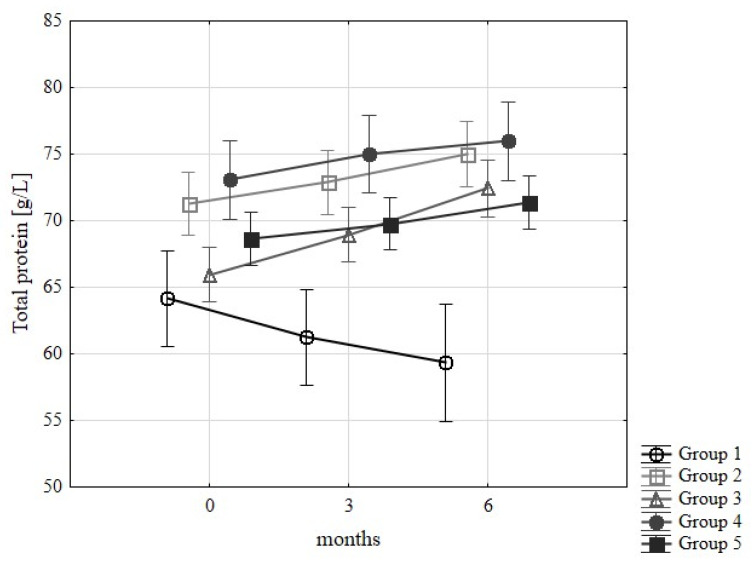
Changes in total protein levels (g/L) in examined groups of patients during 6 months of implementation of diet recommendations.

The total serum protein content of the patient groups is shown in Figure 2, with a range of 62–84 g/L taken as the norm. Its amount was statistically significantly influenced by the following variables: Month of determination and type of group. Statistically significant differences were found between month 0 and month 6 when determinations were made only in group 3. However, the concentration of total protein in the blood of patients in the other groups fed properly increased over time, only decreasing in patients in group 0 until the 6th month of observation.

The mean total protein in group 1 was significantly lower (64.2 ± 6.2 g/L) than in group 4 (73.1 ± 1.8 g/L) at the beginning of the study. At month 3, significance was shown between group 1 (61.3 ± 5.7 g/L) and group 2 (72.9 ± 5.9 g/L), and group 4 (75.0 ± 1.2 g/L), and at month 6 between group 1 (57.9 ± 4.8 g/L) and the others (2- 75.0 ± 4.1 g/L, 3- 72.4 ± 4.0 g/L, 4- 76.0 ± 1.8 g/L, 5- 71.4 ± 3.4 g/L).

Thus, it can be concluded that a proper diet is conducive to maintaining good nutritional status.

### 3.3. Dietary Assessment and Nutritional Value of Diets

The study began with an assessment of the patients’ diets. In the majority of subjects (groups 2–5), it was necessary to modify the diet so that the complete daily ration (CDR) contained the necessary amount of energy and nutrients.

A comparison of the nutritional value of the subjects’ diets before and after starting nutritional therapy is shown in Table 3.

Energy intake in the menu of Group 1 (diet A) was slightly above 60% of the norm. It was statistically significantly lower than the other diets. Energy from glucose in the dialysis fluid covering 30% of energy requirements was not included in the table. However, when this source was included, the calorie supply was within the lower limit of normal.

The low intake of protein in the diet of Group 1 was also shown. The degree of fulfillment of the standard for this component averaged 64.5%. The diets suggested by the dietician (B and C) were characterized by significantly higher protein content.

The CDR of patients in Group 1 showed a low content of almost all the minerals tested, except for sodium. Statistical analysis showed a significantly lower sodium content in their diets than in diet B. Considering the need to limit potassium in hemodialysis patients, an intake of approximately 1700 mg/day could be considered adequate. The amount of the bioelement was significantly lower than that contained in diets B and C. The results of the study also indicate a significant calcium deficiency in diet A. In CDR, calcium content averaged 342 mg against recommendations of 1000–1500 mg/day and was significantly lower than in the other diets. Phosphorus intake was in line with dietary recommendations for people with chronic renal failure. It averaged 775 mg. The values obtained were within the lower limits of the norm, which is 800–1100 mg. Diets B and C yielded statistically significantly higher phosphorus content in CDR.

Regarding the vitamin C content of the diets studied, it was at an average of less than 25 mg in Group 1. The highest amount of vitamin C was found in C, that is, “standard diets enriched with natural antioxidants”.

### 3.4. Blood Concentrations of Vitamin C

Table 4 shows the mean vitamin C concentrations before and after HD in the study groups each month. Mean serum vitamin C concentrations ranged from 8.5 to 17.7 mg/L in the pre-HD samples and from 7.5 to 16.4 mL/L in the post-HD samples (Table 4).

At the start of the study (0 months), the differences in vitamin C concentrations between the groups were statistically insignificant, with the highest values obtained in group 3 (15.7 ± 3 mg/L), where diet C was used. In group 1, there was a decrease in serum ascorbic acid during the 6 months of the study of 5.1 mg/L before HD and 2.9 mg/L after HD, resulting in a decrease of 37.5 and 30%, respectively, which was statistically insignificant. The amount of this antioxidant in group 2 before and after HD was constant. After six months of diet C, group 3 showed a significant increase in ascorbic acid to a value of 17 mg/L; however, the highest significant increase was seen in group 4 (17.7 ± 1.5 mg/L), which received vitamin C supplementation in addition to diet B. In the group of patients with diabetes (group 5), significantly lower antioxidant concentrations were observed compared to group 3 at months 3 and 6 of the study. Furthermore, statistical analysis showed that there were no significant differences between the mean vitamin C concentrations in the following months in groups 2 and 5. It was shown that in group 3, patients on diet C had statistically significantly higher vitamin C concentrations compared to groups 1 and 5 after months 3 and 6 of diet and dialysis therapy.

**Figure 3:**
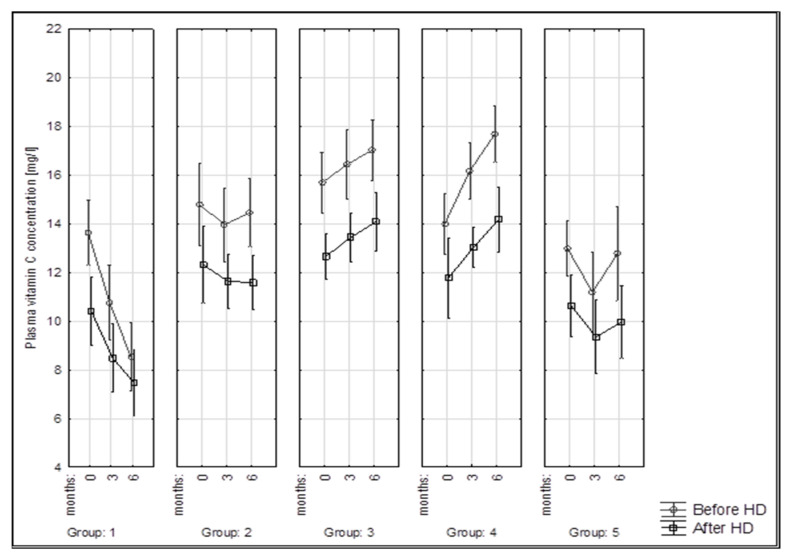
Changes in vitamin C concentration (mg/L) in examined groups of patients during 6 months of the study.

Ascorbic acid concentration after HD was lower in all groups regardless of the month of intake. In groups 2 and 3, a single hemodialysis treatment significantly reduced the amount of this antioxidant at months 3 and 6. Figure 3 aptly illustrates the downward trend in vitamin C content after hemodialysis and the systematic decrease in ascorbic acid concentration in group 1.

### 3.5. Antioxidant Activity of Patients’ Blood Serum

The FRAP index was used to assess the antioxidant activity of the subjects’ serum, with values ranging from 431.1 to 698.4 µM/L in pre-HD samples and from 232.4 to 513.5 µM/L in post-HD samples (Table 5).

The highest beginning antioxidant activity was in group 5 (698.4 ± 86.2 µM/L) (significantly higher than in groups 2, 3, and 4). After 6 months of dietary recommendations, the highest values were obtained in groups 4 (633.8 ± 36.8 µM/L) and 5 (620.6 ± 85.4 µM/L), lower values in groups 3 (612.2 ± 59.1 µM/L) and 2 (556.7 ± 73.2 µM/L), and the lowest values in group 1 (431.1 ± 39.8 µM/L). In group 1, the differences were significantly lower compared to groups 3, 4, and 5.

**Figure 4:**
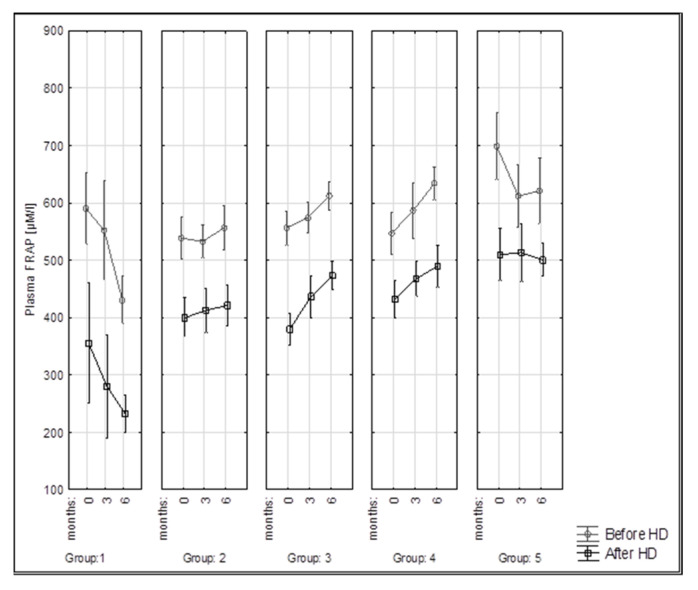
Changes in FRAP activity (µM/L) in examined groups of patients during 6 months of the study.

The dialysis procedure was shown to significantly reduce the reducing capacity of serum in groups 1, 2, and 3 regardless of the month of determination, as well as in group 4 at month 6 and in group 5 at the initial collection (Figure 4).

**Figure 5:**
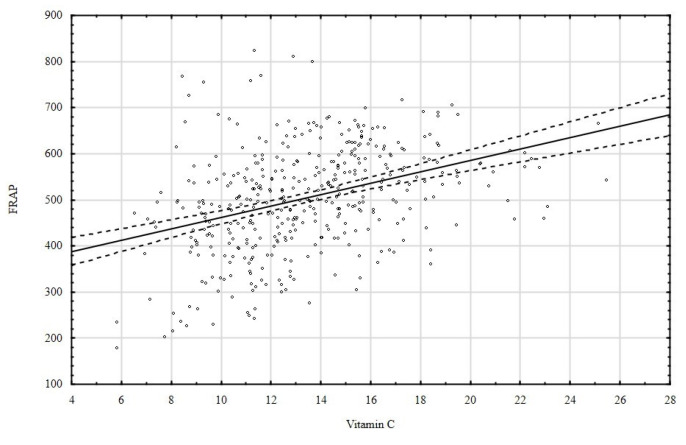
Relationship between vitamin C concentration and FRAP (*r* = 0.36696, 0,95 confidence interval).

A statistically significant positive correlation was observed between the vitamin C concentration and FRAP (correlation coefficient *r* = 0.36696) (Figure 5).

## 4. Discussion

Malnutrition is common among patients on hemodialysis treatment and is usually accompanied by high morbidity and mortality [24,25]. In a significant proportion of patients (20–80%, depending on the source) starting dialysis treatment, nutritional impairment is present, which worsens the course of the disease. This is due to both the course of chronic renal failure and underlying and concomitant diseases and may also be an effect of the treatment undergone.

One of the causes of impaired nutritional status is undoubtedly an unbalanced diet. Among the causes of the reduced nutritional value of the complete daily ration (CDR), the following are most frequently mentioned: The need for an elimination diet (limiting phosphorus, sodium, and potassium intake), an inappropriate diet before starting hemodialysis; the need to comply with a fluid regime; and a lack of appetite [26,27]. The diet used should cover the patient’s energy requirements and provide an adequate amount of macro- and micronutrients. Continuous access of patients (and their families) to a dietician seems to be of significant importance.

The realization of the objectives of the present study began with an assessment of patients’ diets. Our study showed that the diets of patients on hemodialysis were inadequate and that CDR was deficient in macro- and micronutrients and energy.

To maintain the desired body weight, limit gluconeogenesis, and prevent protein catabolism in patients, an adequate energy value of the diet must be ensured. An adequate protein supply is essential to maintaining normal nutritional status. In addition, meals should be small in volume and quantity and their intake should be evenly distributed throughout the day. Too large a single protein supply can result in the accumulation of ammonia in the blood, a product of the urea cycle [28].

Our study showed an inadequate supply of calories and protein in the CDR of the patients participating in the study. The prevalence of these deficiencies in the diets of dialysis patients has been confirmed in many Polish [29,30,31] and foreign studies [32,33,34]. In contrast, Chazot et al. [35], based on a five-year follow-up of the diet of HD patients over 60 years of age, found that energy and protein intake were at recommended levels.

Based on the results of the BMI in our study, it can be concluded that a well-balanced diet can reduce weight loss. During the six-month diet therapy, no weight loss was observed in the adherent groups. A decrease in BMI was noted in Group 1. However, the absolute magnitude of this index remained normal at each stage of the study in all groups.

Some authors emphasize that a BMI < 25 positively correlates with a high risk of increased mortality in this group of patients [36]. Reports in the literature on the magnitude of BMI in this group of patients are divergent. Studies by other authors have observed BMIs between 20 and 25 [37,38,39] and below 20 [40] in patients. In a review of the literature, Aoyagi et al. [41] cite further findings showing a systematic decrease in body weight of chronic dialysis patients over 10–15 years. Confirming the results obtained in our study, Louden et al. [42] showed that an adequate supply of energy and protein in a group of dialysis patients can limit weight loss and even contribute to an increase in BMI. A BMI of over 25 was obtained in the study group.

Considering albumin and total protein concentrations as indicators of nutritional status, the results of our study are promising. The average concentration of albumin and total protein in the subjects was within the middle values of the norm. Only in group 1 was a reduction in their concentration to the lower limit of normal values observed. Similar results were obtained by other authors [38,43]. Mayer et al. [44] indicated albumin levels below 35 g/L and lower-than-normal total protein. The prevalence of low albumin and total protein levels in dialysis patients has been confirmed by other researchers [39,40].

Patients on hemodialysis are a special group of individuals whose leukocytes come into contact with the dialysis membrane, causing them to be stimulated and produce OH¯ and H_2_O_2_ anions exposing them to additional oxidative stress [3,4,5]. In response to the increased production of reactive oxygen species, the body activates mechanisms to protect against oxidative damage. Red blood cells are characterized by a highly effective defense system that includes antioxidant enzymes such as CAT, SOD, and GSH-Px, but also high concentrations of non-enzymatic antioxidants (tocopherols, carotenoids, and vitamin C). The increased release of free radicals also causes partial consumption of substances that form the antioxidant barrier. Vitamin C, which is a water-soluble vitamin, plays a special role in this group, being exposed to oxidants produced by activated neutrophils [45].

Bohm et al. [46], Ha et al. [47], and Wang et al. [48] conducted studies on vitamin C concentrations in hemodialysis patients and showed that, after dialysis treatment, there is a reduction in vitamin C concentrations of approximately 33% to 50% compared to baseline values. Morena et al. [13] found that clearance excretion values for vitamin C vary from 8 to 230 mg/hemodialysis treatment (mean 66 mg/hemodialysis treatment), corresponding to a loss of vitamin C of approximately 200 mg/week, and Bakaev et al. [12] found 132.0 ± 13.6 mg/day [8,9]. Given the high clearance values, the decrease in plasma ascorbic acid concentration during hemodialysis treatment observed in our study is moderate (by 18% on average).

Zwolińska et al. [49] studied the effect of fruit and vegetable restriction in the CRP of dialyzed children on the concentration of vitamins A, E, and C, with simultaneous vitamin C supplementation (100 mg/day). Significantly lower plasma amounts of the aforementioned vitamins were observed compared to healthy subjects without dietary restrictions. In our study, patients who received additional vitamin C were characterized by higher ascorbic acid concentrations. The results obtained are consistent with reports by other authors. Chao et al. [50] began their study by evaluating the diet of both HD patients and a control group of healthy volunteers. They observed that CDR did not differ significantly between the groups, was deficient in energy, and vitamin C was provided in excess. Dietary adjustments were made, and vitamin E and C supplements were introduced (400 mg/after HD for 6 weeks each). At week 6, the vitamin C + E supplemented group significantly increased plasma vitamin C and E levels and plasma antioxidant status compared to the placebo group. After dialysis, the vitamin content did not significantly decrease. In studies by El Mashad et al. [51] and Abdollahzad et al. [52], supplementation with vitamin C at 250 mg/day for 12 weeks significantly increased plasma levels of this vitamin in dialysis patients. Diabetic patients show a reduction in the activity of antioxidant mechanisms, as evidenced by reduced antioxidant concentrations in body fluids and within cells [53]. In addition, patients have been shown to have lower concentrations of the endogenous antioxidants glutathione, ascorbic acid, and tocopherol in plasma, erythrocytes, and platelet cells than healthy controls [54].

Most of the physiological and biochemical mechanisms of action of vitamin C are due to the fact that it is an electron donor, thus exhibiting reducing properties and protecting other cellular components from oxidation. Ascorbic acid can be a two-electron donor. Reversible dissociation of ascorbic acid leads to the formation of the ascorbyl anion, which, by donating one electron, becomes the ascorbyl radical (thus undergoes oxidation). Compared to other free radicals, the ascorbyl radical can be relatively stable, stable, and rather unreactive. These properties make ascorbic acid the preferred antioxidant [55]. The reducing properties of vitamin C determine its antioxidant activity against all RFTs and their derivatives [56]. Ascorbic acid can modulate the redox state of the cell. This is due to both its oxido-reductive properties and its ability to keep other molecules in a reduced state (e.g., the sulfhydryl groups of proteins and glutathione). This allows it to influence many cellular processes regulated by the redox state, such as cell signal transduction, the cell cycle, and DNA repair [57].

No studies on the effect of diet type on the total serum antioxidant capacity of HD patients were found in the available literature.

It was found that the use of balanced diets enriched with supplements or natural antioxidants increased the reducing capacity of the sera of these patients over the 6-month study period.

The results indicate that the serum of patients with a higher L-ascorbic acid content also has a higher antioxidant activity. A statistically significant positive correlation was found between the vitamin C concentration and FRAP. This can be attributed to the strong ability of vitamin C to reduce iron. It reduces Fe^3+^ similarly to hydroxylamine [58] via the following reaction:

ascorbate (AH¯) + Fe^3+^ Fe^2+^ + ascorbyl radical (A^•−^) + H^+^ (1)

Reduced metal ions, e.g., iron, react with hydrogen peroxide leading to the production of highly reactive hydroxyl radicals [59].

The reducing capacity of HD patients’ plasma is not only dependent on diet but also on its content of 35–65% urate, 0–24% ascorbate, 5–10% vitamin E, and 10–50% plasma proteins [60]. The high antioxidant activity may have resulted from uric acid accumulation even in groups that followed dietary recommendations. Studies by other authors indicate increased plasma-reducing capacity in dialysis patients compared to healthy subjects [60,61]. The authors indicated almost twofold higher activity by high uric acid levels. They report that vitamin C supplementation in the dialysis group may account for this disparity. Ahmadpoor et al. [62] obtained a positive correlation between uric acid levels and reducing capacity in hemodialysis patients.

The dialysis procedure was shown to significantly reduce the reducing capacity of serum in groups 1, 2, and 3 regardless of the month of determination, as well as in group 4 at month 6 and in group 5 at the initial collection. Kadkhodaee et al. [61] and Hemmati et al. [63] observed significantly lower antioxidant activity after haemodialysis treatment, which was also shown in our study.

The findings presented in this paper certainly do not reflect the complete oxidative-antioxidant status in the study group. They complement our previous studies, some of whose results have already been published. In a study involving hemodialysis patients with diabetic kidney disease, the activity of the antioxidant enzymes catalase (CAT), superoxide dismutase (SOD), and glutathione peroxidase (GSH-Px) was assessed, and IL-6 and TNF-α levels were determined as an indicator of oxidative stress [64].

## 5. Conclusions

The results of the study indicate the need to monitor nutritional status to prevent malnutrition. The study shows that a well-chosen diet can slow the build-up of malnutrition and increase antioxidant activity in the blood of hemodialysis patients. Vitamin supplements with antioxidant activity can improve the antioxidant status in hemodialysis patients.

## Figures and Tables

**Table 1:** Selected biochemical parameters of patients (mean ± standard deviation SD).

Biochemical Parameters						
Group		1	2	3	4	5
Age (years)		66.2 ± 12.7	57.1 ± 11.3	55.5 ± 12.5	63.4 ± 10.0	71.6 ± 3.1
Sex ratio Female/Male		3/3	9/8	12/13	4/5	5/6
Hematocrit (%)		35.1 ± 3.2	36.3 ± 2.5	37.1 ± 2.0	36.5 ± 5.1	36.8 ± 3.1
Red Blood Cells (×10^12^/L)		14.0 ± 1.6	11.8 ± 2.1	16.2 ± 1.5	13 ± 1.1	17.7 ± 1.5
Uric acid (mg/dL)		6.1 ± 1.1	6.6 ± 1.0	6.5 ± 1.1	6.1 ± 0.8	6.7 ± 0.7
Urea (mg/dL)	Pre-HD	119.7 ± 24.7	149.0 ± 40.0	119.4 ± 27.2	151.1 ± 28.5	134.6 ± 26.1
	Post-HD	48.0 ± 8.4	44.3 ± 7.2	41.3 ± 12.1	47.4 ± 20	46.3 ± 15.1
Creatinine (mg/dL)		9.0 ± 0.6	9.1 ± 0.5	7.0 ± 1.2	8.6 ± 1.0	7.6 ± 0.3

**Table 2:** Body mass index BMI (kg/m^2^) in patients in each group before and after nutritional therapy (mean ± SD).

BMI (kg dry weight/m^2^)			
Months	0	3	6
Group			
1	23.3 ± 3.8 *	23.3 ± 3.9	22.8 ± 4.3 *
2	24.8 ± 4.1 *	24.9 ± 4.0	25.1 ± 4.0 *
3	25.4 ± 3.8	24.8 ± 3.9	25.4 ± 3.7
4	24.3 ± 5.3	24.2 ± 5.2	24.0 ± 5.3
5	21.6 ± 1.3 *	22.1 ± 1.1	22.6 ± 0.8 *

* statistically significant differences at the level of *p* < 0.05.

**Table 3:** Average energy content and selected components of diets.

Types of Diets	A		B		C	
Components	Mean ± SD	% Norm Realization	Mean ± SD	% Norm Realization	Mean ± SD	% Norm Realization
Energy (kcal)	1440.2 ± 480.2 *	61.6	2413.4 ± 106.5 *	102.9	2484.90 ± 63.9 *	106.0
Protein (g)	51.8 ± 18.4 *	64.5	77.7 ± 3.9 *	96.6	78.7 ± 3.8 *	97.8
Sodium (mg)	1782.5 ± 741.3 *	89.2	2418 ± 236 *	120.9	2435.6 ± 181.1	121.8
Potassium (mg)	1703.3 ± 718.4 *	85.2	1934.7 ± 145.5 *	96.8	2072.6 ± 204.5 *	103.7
Calcium (mg)	342.2 ± 210.8 *	22.8	1338 ± 102.4 *	89.2	1343.9 ± 214.4 *	89.6
Phosphorus (mg)	775 ± 295.9 *	70.5	1069.2 ± 118.8 *	97.2	1107.4 ± 52.7 *	100.7
Vitamin C (mg)	24.9 ± 24.6 *	31.2	71.8 ± 6.75 *	89.8	110.3 ± 10.8 *	137.8

* statistically significant differences at the level of *p* < 0.05.

**Table 4:** Comparison of serum vitamin C concentrations (mg/L) between groups before and after HD during 6 months of the study.

Vitamin C (mg/L)						
Months	0		3		6	
Group	Before HD	After HD	Before HD	After HD	Before HD	After HD
1	13.6 ± 1.3	10.4 ± 1.4	10.8 ± 1.5	8.5 ± 1.3	8.5 ± 1.3	7.5 ± 1.3
2	14.8 ± 3.3	12.3 ± 3.1	14 ± 3	11.6 ± 2.1	14.4 ± 2.7	11.6 ± 2.2
3	15.7 ± 3	12.7 ± 2.2	16.4 ± 3.4	13.4 ± 2.4	17 ± 3	14.1 ± 2.9
4	14 ± 1.6	11.8 ± 2.1	16.2 ± 1.5	13 ± 1.1	17.7 ± 1.5	14.2 ± 1.7
5	13 ± 1.7	10.6 ± 1.9	11.2 ± 2.5	9.3 ± 2.3	12.8 ± 2.9	10 ± 2.2

**Table 5:** Comparison of serum FRAP antioxidant activity (µM/L) between groups before and after HD during 6 months of the study.

FRAP (µM/L)						
Months	0		3		6	
Group	Before HD	After HD	Before HD	After HD	Before HD	After HD
1	590.2 ± 59.8	355.2 ± 99.8	552 ± 82.1	280.1 ± 86	431.1 ± 39.8	232.4 ± 31.3
2	538.6 ± 69.5	399.6 ± 67.4	532.7 ± 55	412.8 ± 75.6	556.7 ± 73.2	421.3 ± 70.8
3	556.2 ± 72.4	379.4 ± 65.5	574.1 ± 64.6	436.8 ± 88.7	612.2 ± 59.1	473.3 ± 60
4	547.1 ± 47.3	432.2 ± 43.2	586.2 ± 63.6	467.7 ± 40.7	633.8 ± 36.8	489.8 ± 47.6
5	698.4 ± 86.2	509.9 ± 67.7	611.1 ± 82.2	513.5 ± 75.2	620.6 ± 85.4	500.5 ± 42.5

## Data Availability

Not applicable.
